# Genome analysis uncovers an inverse correlation between alterations in P21‐activated kinases and patient survival across multiple cancer types

**DOI:** 10.14814/phy2.70192

**Published:** 2025-01-05

**Authors:** Jessie M. Vo, Linh M. La, Ananda V. Anderson, Abdulaziz H. Alanazi, Payaningal R. Somanath

**Affiliations:** ^1^ Clinical and Experimental Therapeutics University of Georgia Augusta Georgia USA; ^2^ Department of Clinical Practice, College of Pharmacy Northern Border University Rafha Saudi Arabia; ^3^ Georgia Cancer Center Augusta University Augusta Georgia USA

**Keywords:** cancer, genetic alterations, metastasis, P21 activated kinase, patient survival

## Abstract

Cancer is a complex disease with profound societal and economic impacts, especially in metastatic cases where treatment challenges arise due to the absence of reliable biomarkers and effective therapies. While P21‐activated kinases (PAKs) play a key role in cancer progression, their potential as predictive markers for metastasis and therapeutic targets has not been fully explored. We hypothesized that genetic alterations in PAK isoforms could be linked to reduced overall patient survival. To investigate this, we used data from the cBioPortal for Cancer Genomics, analyzing several randomized, multicentered phase‐3 clinical trial datasets. The analysis revealed significant genetic alterations in PAK genes, particularly in cancers such as breast, prostate, pancreatic, and lung. Notably, elevated PAK expression was associated with poorer survival outcomes in prostate and breast cancer patients. In pancreatic and lung cancers, although a trend of poorer survival with PAK alterations was observed, it was not statistically significant. Our findings underscore the importance of PAK isoforms as potential biomarkers and therapeutic targets, particularly in metastatic cancers. Further research could lead to improved patient outcomes through targeted interventions aimed at PAK‐related pathways, with PAK serving as a reliable biomarker for the precise diagnosis, monitoring, and personalization of treatment strategies.

## 1 INTRODUCTION

Cancer continues to be a significant global health challenge, placing immense burdens on individuals and healthcare systems alike (Bray et al., 2024). Among the most difficult forms to manage are metastatic cancers, which not only present complex biological mechanisms but also often lack reliable biomarkers for accurate diagnosis, staging, and therapeutic targeting (Zhou et al., 2024). Despite remarkable advancements in cancer treatment over the past decades, metastasis remains the leading cause of cancer‐related mortality, underscoring the urgent need for novel therapeutic strategies and prognostic markers (Shi et al., 2024).

The p21‐activated kinases (PAKs) are a family of six serine/threonine kinases (PAK1‐6) that play key roles in various cellular processes, such as cytoskeletal reorganization, cell motility, survival, and proliferation (Kichina et al., 2010; Somanath et al., 2023). These processes are central to cancer progression, particularly in the context of metastasis (Al‐Azayzih et al., 2015; Baker et al., 2014; Goc et al., 2013; Radu et al., 2014; Somanath et al., 2009; Somanath & Byzova, 2009). Several studies have highlighted the involvement of PAKs in oncogenic signaling pathways and cancer progression, suggesting that aberrant expression or activation of these kinases may promote tumorigenesis and metastatic behavior (Kichina et al., 2010; Somanath et al., 2023). Targeting group‐I PAKs (PAK1‐3) has also been demonstrated to suppress tumor growth and metastasis in prostate (Al‐Azayzih et al., 2016; Najahi‐Missaoui et al., 2020; Verma, Artham, et al., 2020; Verma, Najahi‐Missaoui, et al., 2020) and a variety of other cancer types (Kichina et al., 2010; Somanath et al., 2023). However, there is still a significant knowledge gap regarding whether PAK isoforms could serve as reliable predictive biomarkers for cancer prognosis, particularly concerning patient survival outcomes across different cancer types.

Our study seeks to address this gap by analyzing the relationship between genetic alterations in PAK isoforms and patient survival. Utilizing the extensive resources of the cBioPortal for Cancer Genomics database (Cerami et al., 2012; Ping et al., 2014), we analyzed multicenter phase‐3 clinical trial datasets, ensuring a high level of robustness and generalizability. Advanced statistical analysis using SPSS software allowed us to explore correlations between PAK isoform expression, mutation frequency, and survival outcomes in a variety of cancer types. By integrating these datasets, we aimed to uncover potential patterns that could position PAK isoforms as key players in cancer progression and potential markers for prognosis, particularly in the context of metastatic cancers. Our research not only contributes to the growing body of knowledge on PAKs but also offers insights that could inform future therapeutic strategies aimed at improving cancer patient outcomes.

## 2 MATERIALS AND METHODS

### 2.1 DepMap analysis

The Cancer Dependency Map (DepMap; https://depmap.org/portal/) by the Broad Institute profiles thousands of cancer cell lines to identify gene dependencies using RNA interference (RNAi) and CRISPR‐Cas9 knockout technologies (McFarland et al., 2018). Dependency scores, ranging from 0 to −1, reflect the likelihood that a gene is essential for tumor cell survival, with more negative scores indicating higher dependency. DEMETER2 and Chronos algorithms analyze RNAi and CRISPR data, respectively, with the results visualized as bell‐curve distributions. Each gene is evaluated for its effect on cell viability, the fraction of dependent cell lines, and its classification as either “strongly selective” (indicating selective essentiality in specific cancers) or “common‐essential”, thus providing insights into critical genes for potential therapeutic targeting. We analyzed tumor cell line dependencies on individual PAK genes using the latest DepMap dataset, leveraging CRISPR (Avana) Public 19Q2 and Combined RNAi data (Broad, Novartis, Marcotte). These datasets provided a comprehensive view of gene dependencies, enabling the identification of cancer vulnerabilities associated with PAK isoforms.

### 2.2 cBioportal data analysis

To assess the genetic alterations of the six PAK isoforms (PAK1‐6) in clinical tumor samples, the cBioPortal for Cancer Genomics (https://www.cbioportal.org/) was employed (Cerami et al., 2012; Ping et al., 2014). This platform allowed for the examination of DNA‐level changes and RNA‐level gene expression. For DNA analysis, we utilized 200 curated transcriptomic studies from The Cancer Genome Atlas (TCGA), comprising 62,527 profiled samples. These studies were selected to exclude pediatric cases and overlapping samples, thereby maximizing the robustness of the analysis. Gene expression at the RNA level was also queried using the TCGA dataset across different cancer types. Mutations were excluded from further consideration due to the prevalence of amplifications seen in the correlation plot, where the horizontal axis represented copy‐number alterations and the vertical axis depicted mutation types. An oncoprint was generated to visualize the alterations in PAK isoforms‐6 across five individual cancers and in a summary of all cancer types. Matrix heatmaps were created using SRplot (Tang et al., 2023) to evaluate co‐occurrence relationships of PAK expressions in individual cancers based on cBioPortal datasets.

Survival data were extracted from the TCGA datasets available on cBioPortal and systematically reorganized in an Excel spreadsheet to separate censored and deceased cases. Patient profiles with altered PAK isoforms were compared against those without alterations. Kaplan–Meier survival curves were generated using the SPSS online tool.

### 2.3 Statistical analysis

Statistical analyses were performed using SPSS software v29. The analyses including median and mean survival times, hazard ratios, and log‐rank tests were performed to assess the prognostic significance of PAK alterations. These analyses provided a detailed understanding of how PAK genetic alterations correlate with patient survival outcomes across multiple cancer types.

## 3 RESULTS

### 3.1 Analysis of tumor cell line dependency on PAK isoforms

**FIGURE 1:**
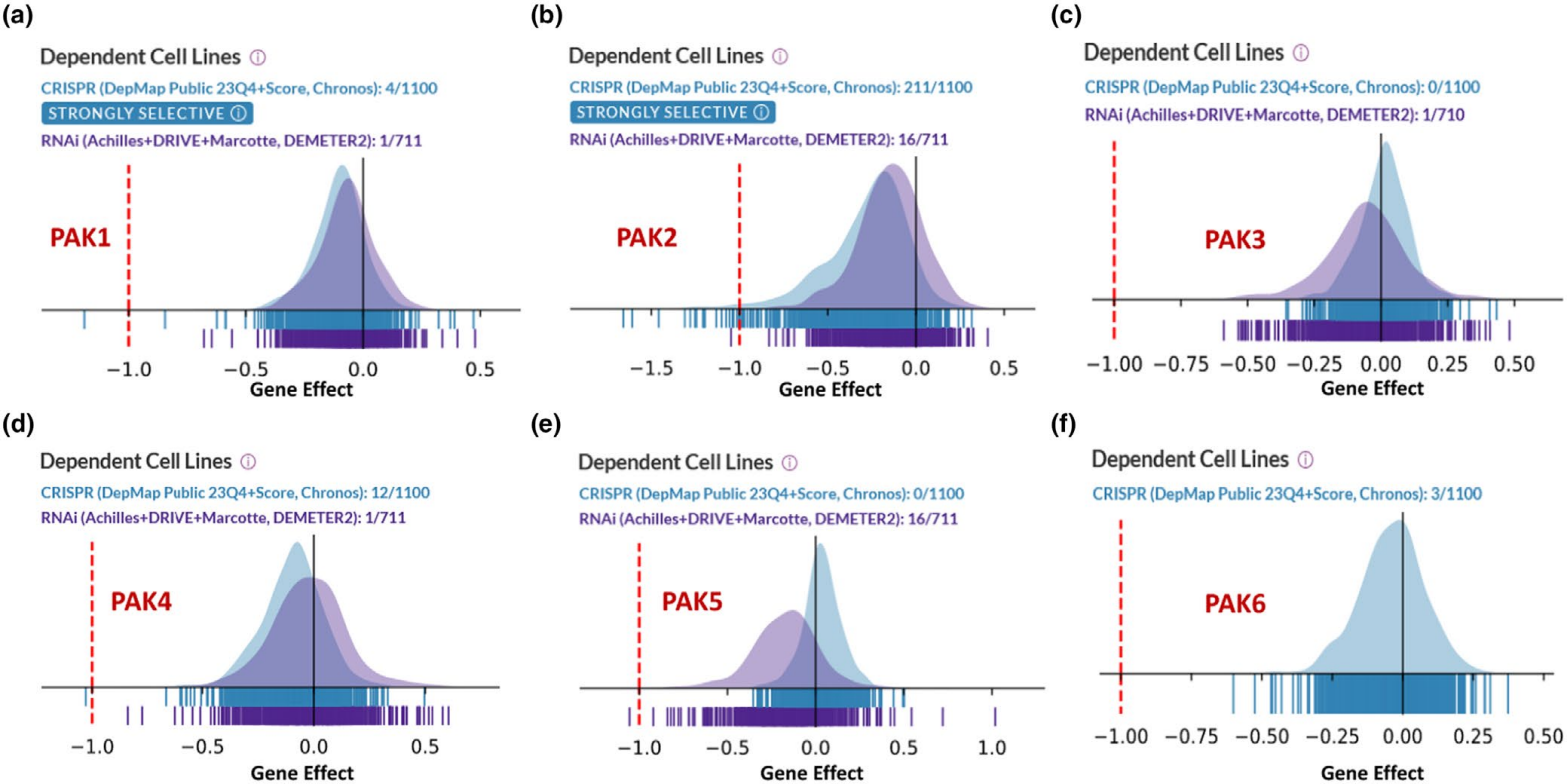
DepMap analysis of the dependency of tumor cell line panels in CRISPR (blue) and RNAi (purple) databases on the indicated PAKS. X‐axis: Gene Effect.

Our data provides a comprehensive comparison of DepMap dependency scores for PAK1, PAK2, PAK3, PAK4, PAK5, and PAK6, based on CRISPR analysis of 1100 tumor cell lines and RNAi analysis of 711 cell lines (Figure 1). Notably, for PAK1, PAK2, and PAK4, CRISPR analysis revealed a higher fraction of dependent cell lines compared to RNAi, while for PAK3 and PAK5, RNAi showed more dependencies. CRISPR knockout also identified PAK1 and PAK2 as strongly selective genes, underscoring their potential as cancer‐specific vulnerabilities (Figure 1). Additionally, no RNAi data was available for PAK6, and none of the six PAK genes were classified as “common essential,” suggesting their roles may be more context‐specific.

### 3.2 Clinical analysis of alterations and expressions in PAK isoforms in cancer

**FIGURE 2:**
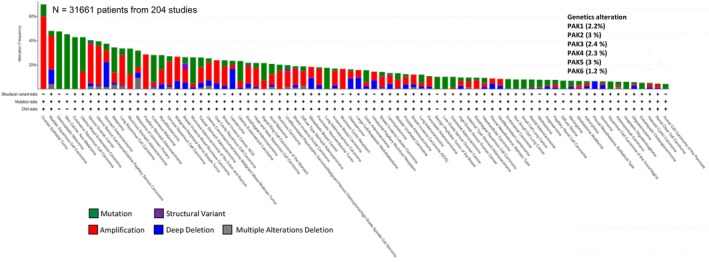
cBioPortal analysis of frequencies of the indicated alterations of PAK1, PAK2, PAK3, PAK4, PAK5, and PAK6 (gene amplification red, deep deletion blue, mutation green) among different cancer subtypes.

In the second phase of our study, we utilized cBioPortal, an open‐source cancer genomics database originally developed by Memorial Sloan Kettering Cancer Center (Cerami et al., 2012; Ping et al., 2014), to assess the genomic alterations in PAK genes across various cancer subtypes. A query was performed across 204 curated studies, encompassing 31,661 tumor samples, to analyze the frequency of alterations in PAK isoforms. Genomic alterations, categorized as mutations (green), fusions (purple), amplifications (red), deep deletions (blue), and multiple alterations (gray), were visualized (Figure 2).

In our analysis, epithelial ovarian tumors exhibited the highest number of overall alterations, with nearly 60% of samples showing gene amplifications, the most common alteration type across all cancers after mutations. Bladder squamous cell carcinoma and desmoplastic melanoma followed, also showing significant levels of PAK alterations, with bladder squamous cell carcinoma characterized predominantly by amplifications. In contrast, desmoplastic melanoma, non‐melanoma skin cancer, and cutaneous squamous cell carcinoma showed alterations limited to mutations (Figure 2).

**FIGURE 3:**
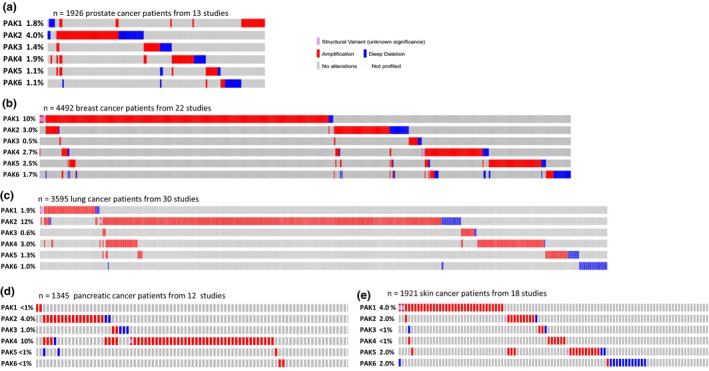
cBioPortal oncoprints showing various alterations in PAK isoforms in different cancers. The percentages on the left‐hand side indicate frequencies of all alterations for the respective PAK.

Next, Oncoprints were generated to detail the alterations in PAK genes across selected cancer types, chosen for their clinical significance rather than alteration frequency (Figure 3). PAK2 was the most frequently altered gene (4%) in prostate cancer (Figure 3a). Similarly, breast cancer displayed a significant enrichment of PAK1 alterations (10%), with other PAKs showing ≤3% alterations (Figure 3b). In lung cancer, PAK2 had the highest incidence of alterations (12%) (Figure 3c), significantly higher than any other PAK isoforms, which ranged from 0.6% to 3% (Figure 3a–e). Interestingly, in pancreatic cancer, PAK4 exhibited the highest frequency of alterations (10%), a notable increase compared to PAK2 (4%) and PAK3 (1%), while PAK1, PAK5, and PAK6 were altered in fewer than 1% of cases (Figure 3d). In skin cancer, which included basal cell carcinoma, cutaneous squamous cell carcinoma, melanoma (including acral, metastatic, and cutaneous subtypes), and desmoplastic melanoma, PAK1 had the highest frequency of alterations (4%), all of which were amplifications (Figure 3e). These findings highlight specific PAK genes as recurrently altered in distinct cancer types, suggesting their potential as biomarkers and therapeutic targets.

**FIGURE 4:**
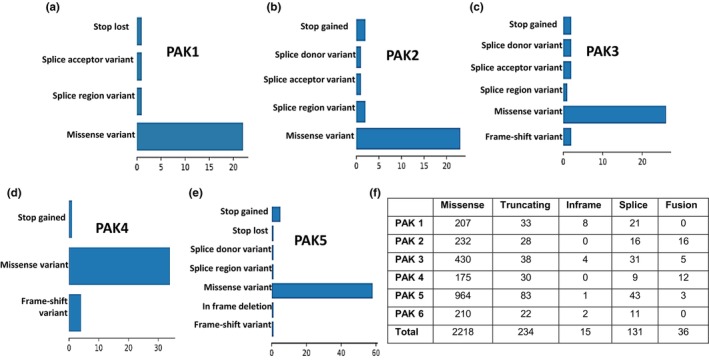
(a–e) Bar graphs displaying the distribution of various mutation types in PAK isoforms across different cancer cell lines, analyzed from DepMap data. (f) Data summarizing the types of mutations in PAK isoforms across multiple cancer types, sourced from cBioPortal.

**FIGURE 5:**
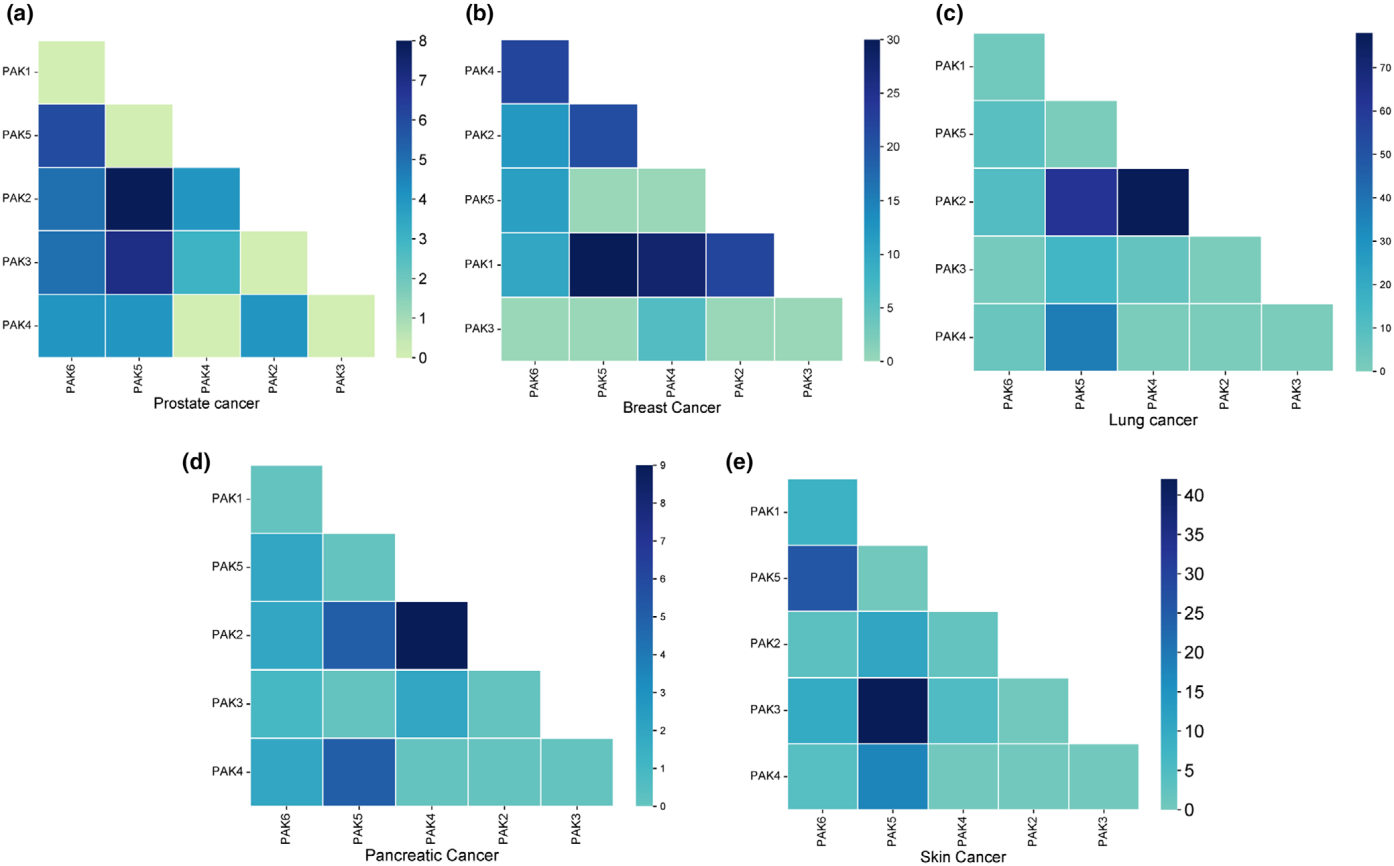
SRplot correlation matrix heatmap showing the extent of correlation between the expression of PAK isoforms in (a) prostate (b) breast (c) lung (d) pancreatic, and (e) skin cancers.

Mutation analysis revealed that the majority of mutations in PAK isoforms are missense in nature (Figure 4), although some splice mutations and truncations were also observed (Figure 4f). To explore the molecular interactions among PAK isoforms, a correlation analysis was conducted to evaluate the associations between gene expression levels. Most PAK isoforms were found to exhibit a positive correlation with statistically significant *p*‐values across five analyzed cancer types (Figure 5). Interestingly, none of the PAK isoforms in prostate (Figure 5a), breast (Figure 5b), lung (Figure 5c), pancreatic (Figure 5d), or skin (Figure 5e) cancers were found to be mutually exclusive. A detailed analysis is included in Table S1.

### 3.3 Analysis of survival curves for patients with alterations in PAK isoforms in cancer

**FIGURE 6:**
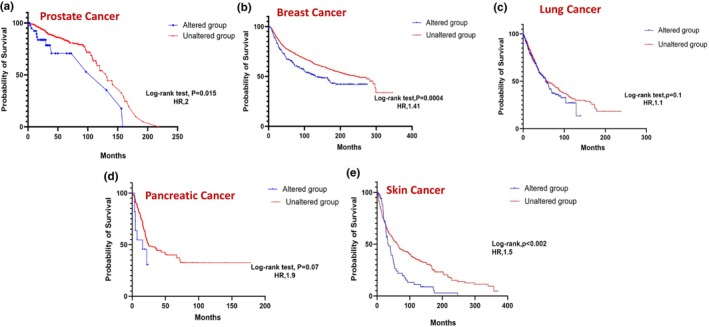
Kaplan–Meier plots correlating PAK1‐6 expression with a probability of survival in (a) prostate (b) breast (c) lung (d) pancreatic, and (e) skin cancers.

We next examined the correlation between PAK isoform expression and patient survival across various cancers. Using cBioPortal data, Kaplan–Meier survival analyses were performed with SPSS Statistics to compare overall survival between patients with altered PAK expression and those with unaltered expression. The presented curves illustrate the probability of survival in months, comparing the altered group (blue) to the unaltered group (red), further highlighting the survival impact of specific PAK alterations in distinct cancer types. Table 1 summarizes the results, showing the number of samples (*n*) and the time to overall survival for each cancer type indicating cancers where PAK isoform expression was both clinically and statistically significant. Overall, significant differences in survival were observed in prostate, breast, and skin cancers (Figure 6a,b,e). Despite a trend in reduced survival rates in lung and pancreatic cancer patients, the data were not significant (Figure 6c,d).

**TABLE 1:** Time to overall survival by cancer type highlighting clinically and statistically significant PAK isoform expression.

Cancer type/gene		Lung	Skin	Prostate	Pancreatic	Breast
All Paks	HR	1.1	1.5	2	1.9	1.41
	Logrank P	0.1	<0.002	0.015	0.07	0.0004
PAK 1	HR	1.3	1.6	3.8		1
	Logrank P	0.2	<0.02	<0.001		0.2
PAK 2	HR	1	1.9	1.2	2.7	1.1
	Logrank P	0.9	0.1	0.6	<0.0001	0.2
PAK 3	HR	0.8	0.8	1.7	0.6	0.9
	Logrank P	0.8	0.8	0.4	0.2	0.8
PAK 4	HR	1	1.7	2.2	3.4	1
	Logrank P	0.7	0.1	0.2	0.001	0.6
PAK 5	HR	0.8	1.2	5.1		1.3
	Logrank P	0.6	0.5	0.01		0.06
PAK 6	HR	1.4	1.7	1		0.8
	Logrank P	0.2	0.07	0.7		0.7

*Note*: Red = statistically significant hazard ratio. Blue = Hazard ratio of 1 (no difference in survival) with a non‐statistically significant *p* value, indicating the probability of a type II error.

**FIGURE 7:**
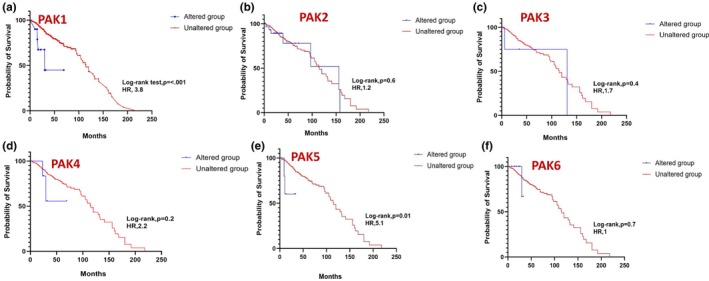
Kaplan–Meier plots correlating each PAK expression with a probability of survival in the case of prostate cancer.

**FIGURE 8:**
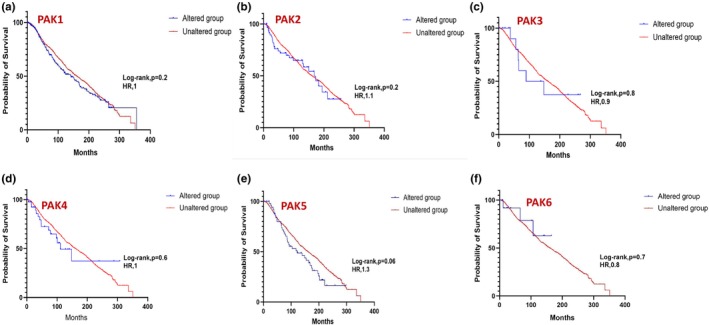
Kaplan–Meier plots correlating each PAK expression with a probability of survival in the case of breast cancer.

**FIGURE 9:**
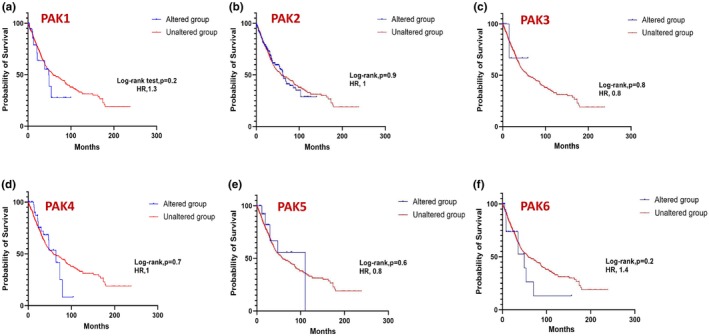
Kaplan–Meier plots correlating each PAK expression with a probability of survival in the case of lung cancer.

**FIGURE 10:**
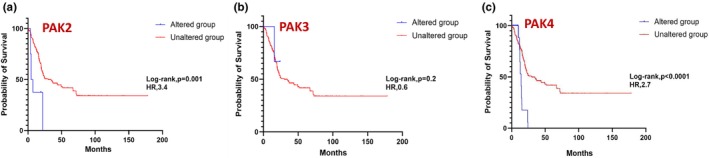
Kaplan–Meier plots correlating PAK2, PAK3, and PAK4 expression with probability of survival in the case of pancreatic cancer.

**FIGURE 11:**
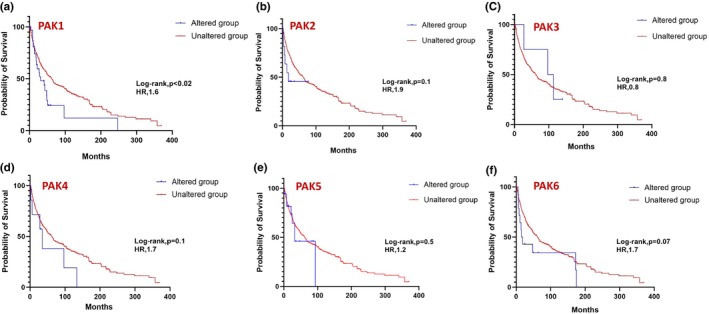
Kaplan–Meier plots correlating each PAK expression with a probability of survival in the case of skin cancer.

In prostate cancer, expressions in PAK1 (Figure 7a) and PAK5 (Figure 7e) showed a statistically significant impact on survival, whereas other PAK isoforms were not observed to be altered in prostate cancer. While individual PAKs did not reach statistical significance in breast and lung cancers, a strong trend in PAK5, similar to prostate cancer was also observed in breast cancer patient survival (Figures 8 and 9). In pancreatic cancer, PAK2 and PAK4 alterations were linked to significant differences in survival outcomes (Figure 10). The data on other PAK isoforms in pancreatic cancer was unavailable. In skin cancer, PAK1 expression was significantly associated with reduced survival (Figure 11), while no correlation was observed between other PAK isoforms and patient survival in skin cancer.

## 4 DISCUSSION

Our comprehensive analysis of PAK isoform dependencies, alterations, and their impact on patient survival across a range of cancers highlights the significant and context‐specific roles of PAK1 and PAK2, along with other PAK family members. Consistent with previous studies, we identified PAK1 and PAK2 as selective and cancer‐specific vulnerabilities, underscoring their potential as key drivers of tumorigenesis and cancer progression (Lei et al., 2022; Somanath et al., 2023). The distinct dependency profiles observed in CRISPR and RNAi analyses, combined with clinical genomic data, emphasize the nuanced roles that different PAK isoforms play in various cancer subtypes. The selective dependencies on PAK1 and PAK2, as revealed by DepMap CRISPR analysis, position these isoforms as critical components of cancer cell survival in specific contexts. PAK1, which has been extensively documented as a mediator of cell motility, proliferation, and survival in various cancers (Al‐Azayzih et al., 2015, 2016; Goc et al., 2013; Kichina et al., 2010; Najahi‐Missaoui et al., 2019, 2020; Somanath et al., 2009, 2023; Somanath & Byzova, 2009; Verma, Artham, et al., 2020), showed higher dependency scores in our study. PAK2, though less studied, displayed a similarly significant dependency, suggesting it may also serve as a promising therapeutic target. Interestingly, the differences observed between CRISPR and RNAi dependency profiles for certain PAK isoforms, such as PAK3 and PAK5, might reflect compensatory mechanisms or off‐target effects that are not apparent in CRISPR knockout models. Further functional studies will be needed to validate these observations and better delineate the context‐dependent roles of PAK isoforms in cancer.

Our clinical analysis of PAK genomic alterations provided further insights into the cancer‐specific relevance of these isoforms. Amplifications in PAK1 and PAK2 were particularly prevalent in breast, lung, prostate, and pancreatic cancers, reinforcing their roles in tumorigenesis. PAK1 amplifications, for instance, have been associated with poor prognosis, chemoresistance, and increased metastatic potential in breast cancer (Belli et al., 2023; Skjervold et al., 2023). Similarly, PAK2 amplifications in lung (Yi et al., 2022) and prostate cancers (Goc et al., 2013; Somanath et al., 2023) suggest its involvement in dysregulated growth factor signaling pathways, such as the PI3K/AKT and MAPK pathways, which are commonly activated in these malignancies. In contrast, PAK3, PAK5, and PAK6 showed fewer alterations and did not exhibit strong associations with clinical outcomes in most cancer types, suggesting that their roles may be more tissue‐specific or context‐dependent. Nevertheless, the deep deletions observed for PAK5 and PAK6 in cancers such as pancreatic and prostate raise the possibility that these isoforms may function as tumor suppressors in certain contexts as has been suggested in hepatocellular carcinoma (Liu et al., 2015), a hypothesis that warrants further investigation. The correlation data suggest that PAK isoforms may function cooperatively rather than independently in the progression of these cancer types. The lack of mutual exclusivity further highlights the potential for synergistic rather than redundant roles of PAK isoforms in tumor biology.

Survival analysis of patients with altered PAK isoform expression reinforced the clinical significance of PAK1 and PAK2. Altered PAK1 expression was associated with reduced survival in skin and prostate cancers, while PAK2 alterations were linked to poor outcomes in pancreatic cancer. These findings align with previous reports that implicate PAK overexpression in aggressive cancer phenotypes, including increased invasion, metastasis, and resistance to therapy (Kichina et al., 2010; Liu et al., 2021; Radu et al., 2014). The significant association between PAK1 and worse survival in skin cancer, as well as the trend toward reduced survival in lung and breast cancers, suggests that PAK1 could serve as a valuable prognostic marker in these malignancies. Similarly, the impact of PAK2 on survival in pancreatic cancer underscores its potential role in driving the aggressive biology of this disease.

One intriguing finding of our study was the presence of structural variants in PAK1, particularly in breast and skin cancers, where these alterations were associated with worse clinical outcomes. Although the functional significance of these structural variants is not yet fully understood, their presence suggests possible alterations in PAK1 protein function or regulation, which could further drive oncogenic processes. Given that structural variants in other kinases have been shown to promote cancer, future research should explore whether PAK1 variants have similar oncogenic potential.

The deep deletions observed in PAK5 and PAK6, particularly in pancreatic and prostate cancers, point to the possibility that these isoforms may act as tumor suppressors. There is not enough supporting literature on PAK5 and PAK6 in pancreatic cancer (Giroux et al., 2009), but the data suggests that these PAK isoforms may play important roles in cellular differentiation and apoptosis. Our finding that PAK5 deletions were associated with poorer survival in prostate cancer further highlights the need for more focused investigations into the functional consequences of these alterations.

This study has several limitations. First, discrepancies between CRISPR and RNAi data reflect potential off‐target effects or incomplete knockdown, requiring further validation. Second, reliance on public genomic databases like cBioPortal may introduce biases due to varying data quality and underrepresentation of certain cancer types. Third, while correlations between PAK alterations and outcomes were identified, the lack of mechanistic studies limits our understanding of causality. Survival analyses may be influenced by confounding factors not fully accounted for. Fourth, our analysis does not separate cases by stage or treatment history, which may hide the significance of specific PAK alterations in early‐stage cancers or those involving therapy resistance, where PAKs might only affect outcomes linked to certain treatment regimens. Fifth, the differences between CRISPR‐ and RNAi‐revealed dependencies may arise from partial protein suppression by RNAi, which can sustain cell survival, and potential CRISPR artifacts, where targeting highly amplified PAK genes causes excessive DNA damage that overwhelms repair mechanisms. Lastly, although many studies have indicated the potential benefits of targeting group I PAKs (PAK1‐3) for cancer therapy (Al‐Azayzih et al., 2015; Kichina et al., 2010; Rudolph et al., 2013; Semenova & Chernoff, 2017; Somanath et al., 2023; Verma, Artham, et al., 2020; Verma, Najahi‐Missaoui, et al., 2020), clinical validation of other PAK isoforms as therapeutic targets is needed to confirm their potential in cancer treatment.

In summary, our study provides a detailed characterization of PAK isoform dependencies, alterations, and their clinical relevance across diverse cancer types. PAK1 and PAK2 emerge as critical regulators of cancer progression and survival, making them compelling targets for therapeutic development. Future research should aim to elucidate the precise molecular mechanisms through which these isoforms drive oncogenesis, particularly in the context of their interactions with key signaling pathways. Additionally, the role of structural variants in PAK1 and the potential tumor‐suppressor functions of PAK5 and PAK6 represent important areas for future exploration. Understanding these dynamics will be crucial for optimizing the use of PAK‐targeted therapies and identifying new biomarkers for cancer prognosis and treatment response.

## FUNDING INFORMATION

The Kenneth L. Waters Foundation Endowment and National Institutes of Health grant UL1TR002378 to PRS.

## CONFLICT OF INTEREST STATEMENT

P.R.S. is the Co‐Founder and Chief Scientific Officer of MetasTx LLC.; other authors declare no conflict of interest.

## ETHICS STATEMENT

This study was conducted using the publically available datasets thus exempted from IRB approvals.

## Supporting information


Table S1.

## Data Availability

The datasets used and/or analyzed in the current study are available from the corresponding author upon reasonable request.
