# Supplementary material for: Genome analysis uncovers an inverse correlation between alterations in P21‐activated kinases and patient survival across multiple cancer types
Source: Physiol Rep. 2025 Jan 5;13(1):e70192. doi: 10.14814/phy2.70192 (PMC11702381; doi:10.14814/phy2.70192)
Supplement: Supplementary file 1 — Table S1. [file PHY2-13-e70192-s001.docx]

**Supplemental Table 1:** Co-expression of PAK isoforms in cancer

| A | B | Neither |  | A not B | B not A | Both | Log2 Odds Ratio | p-Value |
| --- | --- | --- | --- | --- | --- | --- | --- | --- |
|  |  |  |  |  | **Prostate** |  |  |  |
| PAK3 | **PAK5** | 5077 |  | 40 | 42 | 7 | >3 | <0.001 |
| PAK1 | **PAK4** | 5073 |  | 41 | 45 | 7 | >3 | <0.001 |
| PAK5 | **PAK6** | 5063 |  | 43 | 54 | 6 | >3 | <0.001 |
| PAK2 | **PAK5** | 4961 |  | 156 | 41 | 8 | 2.633 | <0.001 |
| PAK3 | **PAK6** | 5064 |  | 42 | 55 | 5 | >3 | <0.001 |
| PAK1 | **PAK2** | 4961 |  | 41 | 157 | 7 | 2.432 | <0.001 |
| PAK4 | **PAK5** | 5069 |  | 48 | 45 | 4 | >3 | <0.001 |
| PAK4 | **PAK6** | 5058 |  | 48 | 56 | 6 | 2.912 | <0.003 |
| PAK2 | **PAK5** | 4961 |  | 158 | 41 | 6 | 2.2 | <0.003 |
| PAK3 | **PAK4** | 5070 |  | 44 | 49 | 3 | 2.819 | <0.001 |
| PAK2 | **PAK6** | 4947 |  | 159 | 55 | 5 | 1.5 | <0.041 |
|  |  |  |  |  | **Breast** |  |  |  |
| PAK4 | **PAK6** | 5815 |  | 152 | 87 | 22 | >3 | <0.001 |
| PAK2 | **PAK5** | 5769 |  | 177 | 109 | 21 | 2.651 | <0.001 |
| PAK5 | **PAK6** | 5848 |  | 119 | 98 | 11 | 2.464 | <0.001 |
| PAK1 | **PAK4** | 5478 |  | 424 | 146 | 28 | 1.309 | <0.001 |
| PAK2 | **PAK6** | 5781 |  | 186 | 97 | 12 | 1.943 | <0.001 |
| PAK4 | **PAK5** | 5784 |  | 162 | 118 | 12 | 1.860 | <0.001 |
| PAK1 | **PAK5** | 8595 |  | 808 | 149 | 30 | 1.099 | <0.001 |
| PAK3 | **PAK4** | 5861 |  | 41 | 168 | 6 | 2.352 | <0.002 |
|  |  |  |  |  | **Lung** |  |  |  |
| PAK2 | **PAK4** | 5339 |  | 644 | 169 | 78 | 1.936 | <0.001 |
| PAK1 | **PAK5** | 36477 |  | 829 | 1332 | 65 | 1.102 | <0.001 |
| PAK4 | **PAK5** | 5627 |  | 210 | 356 | 37 | 1.478 | <0.001 |
| PAK1 | **PAK4** | 5795 |  | 188 | 230 | 17 | 1.188 | <0.003 |
| PAK2 | **PAK5** | 5177 |  | 660 | 331 | 62 | 0.555 | <0.009 |
|  |  |  |  |  | **Pancreatic** |  |  |  |
| PAK2 | **PAK5** | 1435 |  | 20 | 4 | 5 | >3 | <0.001 |
| PAK2 | **PAK4** | 1390 |  | 16 | 49 | 9 | >3 | <0.001 |
| PAK4 | **PAK5** | 1402 |  | 53 | 4 | 5 | >3 | <0.001 |
| PAK5 | **PAK6** | 1449 |  | 7 | 6 | 2 | >3 | <0.001 |
| PAK2 | **PAK6** | 1433 |  | 23 | 6 | 2 | >3 | <0.007 |
| PAK4 | **PAK6** | 1400 |  | 56 | 6 | 2 | >3 | <0.037 |
| PAK3 | **PAK4** | 1399 |  | 7 | 56 | 2 | 2.835 | <0.046 |
| PAK3 | **PAK6** | 1448 |  | 8 | 7 | 1 | >3 | <0.048 |
|  |  |  |  |  | **Skin** |  |  |  |
| PAK3 | **PAK5** | 1836 |  | 45 | 323 | 42 | 2.407 | <0.001 |
| PAK5 | **PAK6** | 1832 |  | 339 | 49 | 26 | 1.520 | <0.001 |
| PAK3 | **PAK6** | 2094 |  | 77 | 65 | 10 | 2.065 | <0.001 |
| PAK4 | **PAK5** | 1840 |  | 41 | 347 | 18 | 1.219 | <0.006 |
| PAK1 | **PAK6** | 2086 |  | 85 | 67 | 8 | 1.551 | <0.011 |
| PAK2 | **PAK3** | 2098 |  | 61 | 80 | 7 | 1.589 | <0.015 |
